# The Ticking of the Epigenetic Clock: Antipsychotic Drugs in Old Age

**DOI:** 10.3389/fendo.2016.00122

**Published:** 2016-08-31

**Authors:** Adonis Sfera, Carolina Osorio, Luzmin Inderias, Michael Cummings

**Affiliations:** ^1^Psychiatry, Patton State Hospital, Patton, CA, USA; ^2^Psychiatry, Loma Linda University, Loma Linda, CA, USA

**Keywords:** antipsychotic medications, stroke, VEGF, microRNA-29, SIRT-1, diabetes mellitus

## Abstract

**Background:**

Exposed to antipsychotic drugs (APDs), older individuals with dementing illness are at risk of cerebrovascular adverse effects (CVAE), including sudden death. Transient microvascular dysfunctions are known to occur in younger persons exposed to APDs; however, they seldom progress to CVAE, suggesting that APDs alone are insufficient for engendering this untoward effect. It is, therefore, believed that a preexistent microvascular damage is necessary for CVAE to take place, but the exact nature of this lesion remains unclear. CNS small vessel disease (SVD) is a well-known age-related risk factor for strokes, dementia, and sudden death, which may constitute the initial CVAE-predisposing pathology. Therefore, we propose the two strikes CVAE paradigm, in which SVD represents the first strike, while exposure to APDs, the second. In this model, both strikes must be present for CVAE to take place, and the neuroimaging load of white matter hyperintensities may be directly proportional with the CVAE risk. To investigate this hypothesis at the molecular level, we focused on a seemingly unrelated phenomenon: both APDs and SVD were found protective against a similar repertoire of cancers and their spread to the brain (1–4). Since microRNA-29 has shown efficacy against the same malignancies and has been associated with small vessels pathology, we narrowed our search down to this miR, hypothesizing that the APDs mechanism of action includes miR-29 upregulation, which in turn facilitates the development of SVD.

**Aim:**

To assess whether miR-29 can be utilized as a peripheral blood biomarker for SVD and CVAE risk.

**Method:**

We conducted a search of experimentally verified miR-29 target genes utilizing the public domain tools miRanda, RNA22 and Weizemann Institute of Science miRNA Analysis. We identified in total 67 experimentally verified target genes for miR-29 family, 18 of which correlate with microvascular integrity and may be relevant for CVAE.

**Conclusion:**

Upregulated microRNA-29 silences the expression of 18 genes connected with capillary stability, engendering a major vulnerability for SVD (first strike) which in turn increases the risk for CVAE after exposure to APDs (second strike).

## Introduction

Cerebrovascular adverse effects (CVAE) of antipsychotic drugs (APDs) were first noted in dementia clinical trials in 2001 (5, 6). In 2004, the United Kingdom Committee on the Safety of Medicines issued a warning to physicians regarding the potential risk for stroke in elderly with dementia treated with risperidone or olanzapine. In 2005, the US Food and Drug administration undertook a meta-analysis of over 5,000 patients with dementia-related psychosis and concluded that aripiprazole, olanzapine, quetiapine, and risperidone may be associated with CVAE (7). A Medicare cohort study, looking at typical and atypical antipsychotics in over 22,000 patients age 65 and older, concluded that conventional antipsychotics were associated with a 37% higher mortality due to CVAE than the atypical ones (8). A recent study revised upward the risk of death in older adults with dementing illness treated with APDs (9).

Cerebrovascular adverse effects are rare events, which occur in older individuals with dementia shortly after the initiation of APD treatment. They are believed to be unrelated to the metabolic adverse effects, which develop over an extended period of time, although both may involve similar pathogenesis (10).

**Figure 1:**
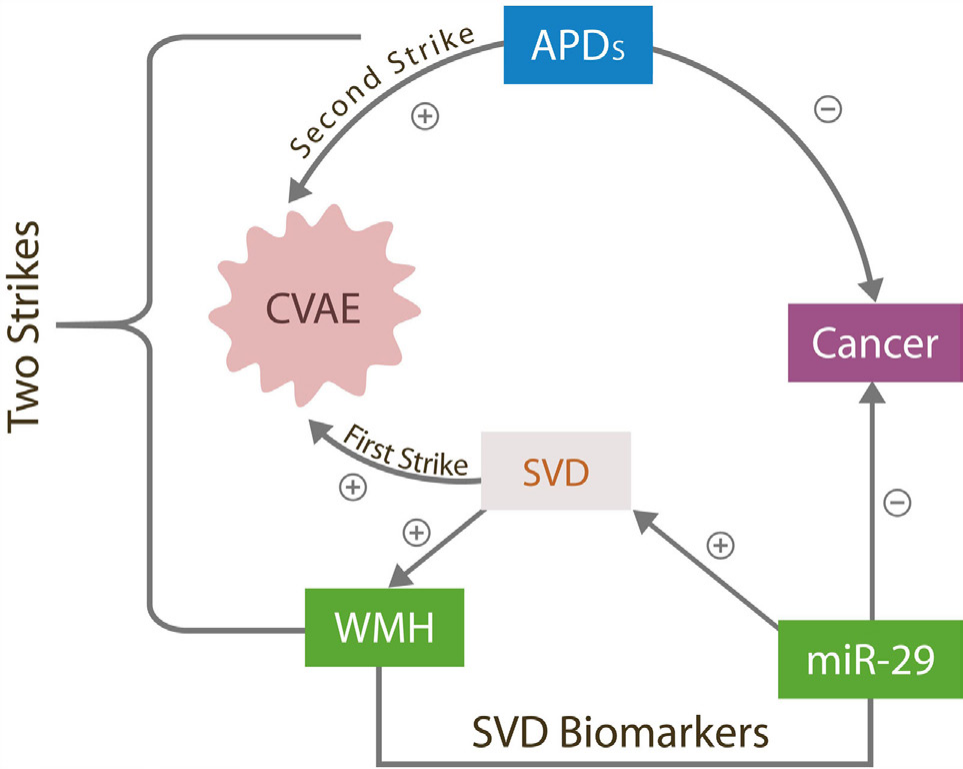
**The two strikes CVAE hypothesis**. The antitumor effects of overexpressed miR-29 destabilizes cerebral microvessels, contributing to SVD, the first CVAE strike manifested by WMHs. APD, antipsychotic drugs; CVAE, cerebrovascular adverse effects; SVD, small vessels disease; WMH, white matter hyperintensities; miR-29, microRNA-29 family.

Transient cerebral small vessels lesions have been reported previously in younger individuals with schizophrenia during the early phase of APD treatment; however, they almost never progress to CVAE, suggesting that the APDs alone are insufficient for triggering these adverse effects (11). A preexistent, occult cerebral small vessel disease (SVD) is a likely predisposing factor as it may engender a background of microvascular instability upon which the addition of APDs could trigger CVAE. In our two strikes model, brain SVD [measured by the white matter hyperintensities (WMHs load)] comprises the first strike, while exposure to APDs the second. CVAE can only occur in the presence of both strikes (Figure 1).

Several studies have demonstrated that cerebral SVD alone increases the risk for strokes, cognitive impairment, and sudden death (12, 13). In addition, this condition is often associated with hypertension, atherosclerosis, or diabetes mellitus (DM), disorders known to induce CNS microvascular lesions (14). Novel epigenetic studies have reported upregulation of microRNA-29 in some cancers, DM, atherosclerosis, hypercholesterolemia, and normal aging, while downregulation of this miR characterizes Alzheimer’s disease (AD) (15–17).

**Figure 2:**
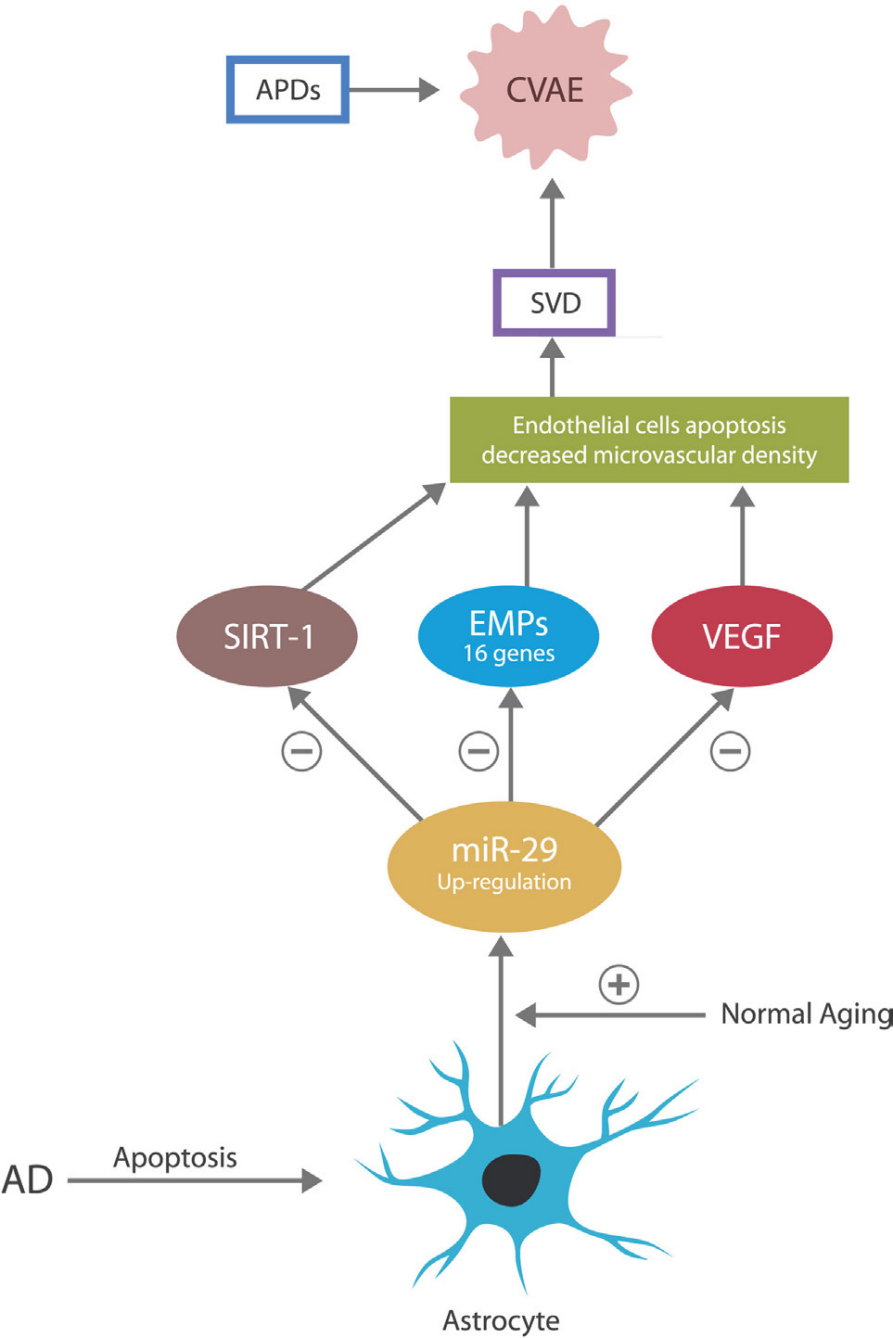
**In the CNS, the astrocyte is the source of miR-29**. In normal aging, miR-29 is overexpressed for neuroprotective purposes. In AD, astrocytes undergo early apoptosis, depressing the levels of miR-29, which may account for the neuronal aberrant entry into the cell cycle and subsequent apoptosis.

Malignancies were demonstrated capable of hijacking microvascular cells and utilize them for tumorigenesis and metastatic spread. They accomplish this through cancer-expressed biomolecules and oncogenes, which alter gene expressions to support cancer-related angiogenesis and microvascular recruitment (18–20). The anti-malignancy action of miR-29 consists of directly silencing these genes, thus blocking the cell cycle entry of both tumor cells and the cells hijacked by tumors (primarily the endothelial cells and pericytes in brain microvessels). This action prevents cancer growth and spread but increases the risk of small vessels damage, as these cells must undergo frequent mitosis in order to maintain the integrity of capillary beds. For example, miR-29 was demonstrated to control the expression of NAD-dependent deacetylase sirtuin-1 (SIRT-1) gene, vascular endothelial growth factor (VEGF) gene, and 16 genes coding for extracellular matrix proteins (EMPs), in charge of small vessels’ integrity. Overexpression of miR-29 may silence these genes, leading to microvascular lesions and SVD (21, 22) (Figure 2).

**Figure 3:**
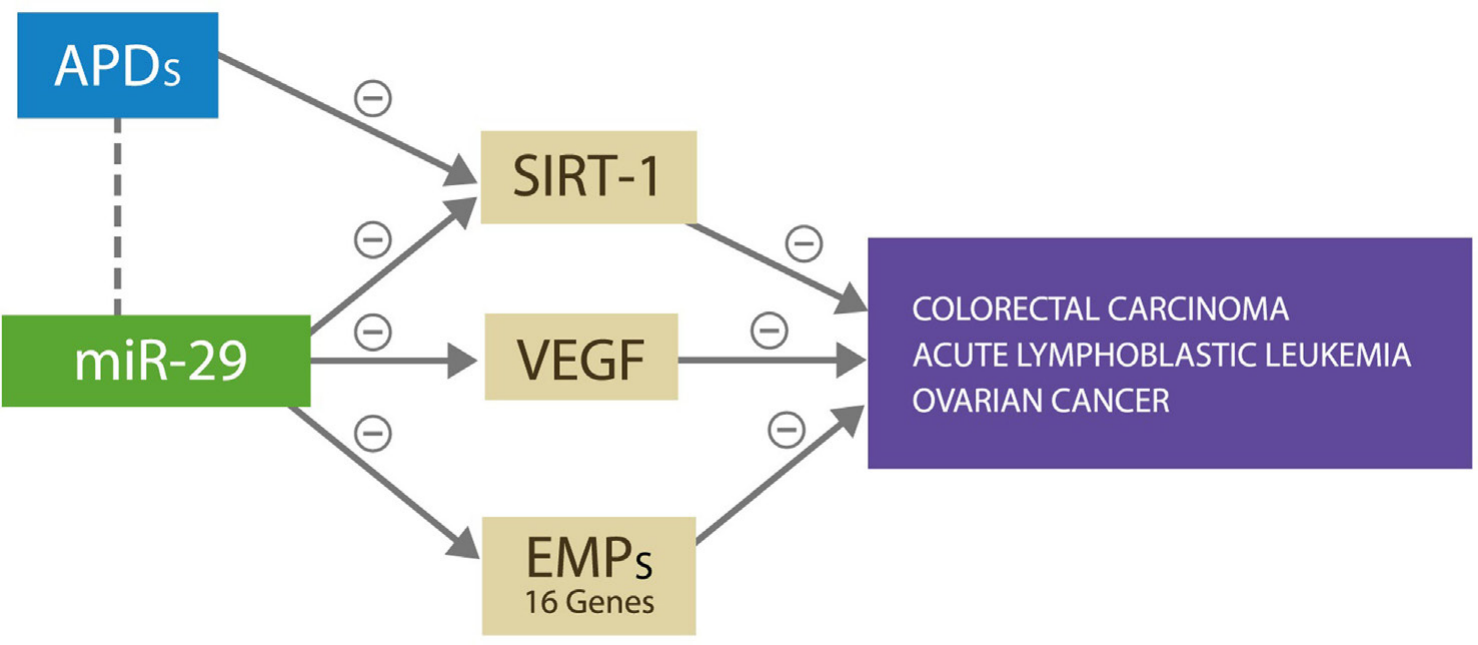
**miR-29 silences SIRT-1, VEGF, and 16 other genes expressing ECM proteins, preventing cancer growth and metastasis by denying access to microvascular recruitment and angiogenesis necessary for spread**. APDs exert antitumor effect against the same malignancies by silencing the SIRT-1 gene.

Antipsychotic drugs were reported to have therapeutic value in colorectal carcinoma, acute lymphoblastic leukemia, and the ovarian cancer, the same malignancies in which miR-29 was found beneficial (23–26). Furthermore, it was demonstrated that the antitumor actions of APDs rely on silencing the expression of SIRT-1 gene and/or altering DNA methylation, both of which are known properties of miR-29 (27) (Figure 3). Therefore, we suggest that APDs induce miR-29 overexpression, which in turn provides therapeutic benefit in these cancers but at the same time facilitates the development of SVD, the first CVAE strike. In addition, we present two potential biological markers of SVD and by extension of CVAE risk: a neuroimaging hallmark, WMH, and a peripheral blood marker, microRNA-29.

## Cerebral Small Vessel Disease, the First CVAE Strike

Cerebral SVD represents a group of age-related conditions, including deep brain infarcts, lacunar infarcts, microbleeds, and white matter lesions. These disorders have a common denominator: involvement of small CNS arteries, arterioles, capillaries, and small veins, increasing the risk of cognitive impairment and geriatric syndromes (28, 29). Recent data demonstrate that 25–30% of strokes are associated with SVD, but even in the absence of larger cerebrovascular accidents, SVD may result in cognitive decline, representing the most common cause of vascular and mixed dementias. The neuroimaging hallmark of SVD is WMHs frequently visualized on magnetic resonance imaging (MRI) in older persons (30).

Both genetic and epigenetic factors have been involved in the cerebral SVD, and its heritability is estimated at 55–80%, while epigenetic mechanisms described in its pathogenesis include methylation and posttranslational modifications, impairing the cell cycle and causing apoptosis of endothelial cells and pericytes (31, 32). A rare form of familial SVD, indistinguishable from the sporadic form, was described in association with impaired collagen type IV, one of the EMPs directly controlled by miR-29 (33, 34).

A recent, large epidemiologic study in SVD revealed that in elderly, the amount of WMHs on MRI was proportional to the increase in systolic blood pressure (BP) (35). Recent studies demonstrated that BP homeostasis is impacted negatively by a dysfunctional biomolecule, the peroxisome proliferator-activated receptor gamma (PPAR-gamma) found in the microvascular smooth muscle cells (36). Interestingly, PPAR-gamma agonists were recently shown to induce the expression of VEGF gene (a gene directly controlled by miR-29), promoting angiogenesis and tissue repair after myocardial infarctions (37). A recent study found miR-29 downregulation to protect against hypertension-induced myocardial ischemia, while another study demonstrated the involvement of dysfunctional miR-29 in pulmonary hypertension (38, 39). Moreover, miR-29a was associated with age-induced aneurysm formation and was found to be overexpressed in cultured senescent endothelial cells (40, 41). Taken together, these data suggest that miR-29 plays a key role in hypertension-associated pathology, which can lead to SVD.

Antipsychotic drugs were demonstrated to engender transient microvascular impairment in younger individuals with schizophrenia during the early phase of treatment (11). These vascular changes, even though insufficient to trigger CVAE in adults without SVD, suggest an association between APDs and brain microvascular dysfunctions. Indeed, studies in rodents documented cerebral small vessel lesions in APDs-exposed animals (42, 43).

In cancer and AD, microRNA-29 was demonstrated to alter DNA methylation (DNAm), affecting expression of many genes (44, 45). While silencing cancer-related genes, this epigenetic mechanism may also engender microvascular damage (46, 47). Interestingly, some APDs were associated with increased DNAm in various areas of the CNS, suggesting that APDs and miR-29 may share a common mechanism of action. In this respect, a recent preclinical study demonstrates that olanzapine induces cerebellar and hippocampal DNAm, while another study showed that haloperidol upregulates DNAm in the striatum (48, 49). Interestingly, schizophrenia was associated with de-methylation of various gene promoters; a recent twin study documented the hypomethylation of ST6GALNAC1 gene promoter in the schizophrenia-affected twin, suggesting that a DNAm deficit may play a role in the pathogenesis of this disorder (50).

Studies in normal aging describe the “epigenetic clock” as measuring DNAm at specific genomic sites. The amount of DNAm in these areas was found to be highly correlated with the human chronological age (51). Since SVD occurs in older individuals and aging is proportional to DNAm and miR-29 expression, the “epigenetic clock” may measure the degree of microvascular damage. Indeed, in pluripotent stem cells, low levels of miR-29 were shown to induce DNA demethylation, bringing these cells a step closer to the embryonic stem cells (in which DNAm is almost nil) (52).

In regard to miR-29 in AD, an apparent paradox has been described: this miR is overexpressed in normal aging but under-expressed in age-related pathologies, including AD. For example, dementia studies report that AD patients present with low levels of miR-29 and that adding recombinant pre-miR-29b to the therapeutic regimen could be beneficial (15, 53). In addition, several studies demonstrated that miR-29 is neuroprotective and that its loss may, at least in part, account for the pathogenesis of AD (54). However, seen from another angle, the low miR-29 level in AD may not represent a paradox at all. As astrocytes secrete this miR exclusively and astrocytes are known to undergo early apoptosis in AD, the low levels of this miR may simply reflect the loss of its source (55) (Figure 2).

In addition, miR-29 is a known blocker of cellular entry into the cell cycle, and its protective role in the brain may include preventing the neurons, from attempting division. Indeed, in AD, neurons were demonstrated to attempt cell cycle entry in an aberrant manner, undergoing apoptosis as they lack the cellular machinery to complete this process (56, 57). Preclinical studies demonstrate that blocking miR-29 induces post-mitotic cells (including adult cardiomyocytes) to engage aberrantly in division (58). Indeed, one study revealed that the unopposed cyclin D2, a direct target of microRNA-29a, induces post-mitotic cells to attempt mitosis (59). We surmise that the early loss of astrocytes in AD accounts for miR-29 depletion, which in turn disinhibits cyclin D2, triggering neuronal apoptosis following unsuccessful attempts at completing the cell cycle.

## CVAE and Metabolic Adverse Effects of APDs: Same Pathogenesis, Different Timing?

In older individuals with dementia, CVAE are rare untoward events associated with the early phase of treatment with APDs. In spite of the fact that they are considered unrelated to the metabolic adverse effects, which are known to occur later in the treatment, they may share an identical pathogenesis.

It is well known that the incidence of cancer, metabolic disorders, and dementias increases with age. As these conditions frequently affect the CNS capillary bed, the genes associated with cerebral microvascular integrity may play a crucial role in their pathogenesis. Moreover, since upregulation of micro-RNA-29 occurs in normal aging and this miR blocks the expression of SIRT-1, VEGF, and several EMPs, it may be crucial to these pathologies.

Several new studies demonstrated that SIRT-1 gene plays a key role in the energy balance, as it functions as a “metabolic sensor,” modulating cellular responses to the energy availability (60). As some APDs silence SIRT-1 gene, this gene may represent the missing link between the CVAE pathogenesis and that of metabolic adverse effects of APDs. Indeed, it was demonstrated that SIRT-1, a NAD^+^-dependent histone deacetylase, regulates the glucose and lipid metabolism by deacetylating several key metabolic effectors (61). Silencing the expression of SIRT-1 was demonstrated to result in insulin resistance, type 2 diabetes mellitus (T2DM), and dyslipidemias, which are all recognized adverse effects of atypical APDs (17, 62). In addition, blocking the expression of VEGF was associated with hypercholesterolemia and insulin resistance (63–65). As miR-29 upregulation accompanies the same metabolic disorders, it suggests that (1) miR-29 overexpression triggers metabolic disorders by silencing SIRT-1 and VEGF genes and (2) APDs upregulate miR-29 as part of their mechanism of action, thus enabling the development of metabolic adverse effects.

In addition to regulating glucose metabolism at the level of insulin-target cells, preclinical studies documented that VEGF and SIRT-1 genes act directly on pancreatic beta cells, facilitating insulin release (66, 67). Moreover, a preclinical study demonstrated a direct link between miR-29 and pancreatic insulin release, further connecting this miR with the metabolic adverse effects of APDs (68). Furthermore, miR-29-induced cell cycle blockade may affect negatively the blood–brain barrier (BBB), especially at the level of endothelial cells and pericytes. As these cells express glucose transporters that help shuttle glucose to the brain, their dysfunction would interfere with this process, further impairing the CNS metabolism (69). In addition to metabolic disorders, breaches of the BBB were associated with increased risk of SVD, stroke, and AD (70–72). Furthermore, since both pericytes and endothelial cells were demonstrated to secrete VEGF, a dysfunction of these cells may impair VEGF synthesis and result in further microvascular damage (18, 73).

Metabolic disorders, including T2DM, were found comorbid with several cancers among which are the acute lymphoblastic leukemia and colorectal and ovarian cancer, the same malignancies in which miR-29 and APDs provide therapeutic benefit, suggesting yet another link between metabolic disorders, APDs and miR-29 (74–76) (Figure 2). Moreover, in colorectal carcinoma chlorpromazine, clozapine and olanzapine were documented to inhibit SIRT-1 gene expression, a gene directly controlled by miR-29, and in acute lymphoblastic leukemia, phenothiazines were shown to block T cells’ division, utilizing a property of miR-29. These data suggest once more that miR-29 may be upregulated by APDs with metabolic and microvascular consequences (43) (Table 1). Interestingly, miR-29c was found to be downregulated in the prefrontal cortex of patients with schizophrenia (77, 78).

**Table 1:** **The beneficial effect of antipsychotic drugs and microRNA-29 by cancer type with references**.

Antipsychotic drugs (APDs)	Cancer type	References APDs	References microRNA-29
Chlorpromazine	Colorectal	(26)	(2)
Clozapine/olanzapine	Non-small cell lung	(23)	(79)
Phenothiazines	Acute lymphoblastic leukemia	(25)	(4)
Thioridazine	Ovarian	(24)	(3)

Recent studies demonstrated that miR-29 has the capability of blocking melatonin secretion from the pineal gland by binding to melatonin-1 receptors. As melatonin is a powerful inhibitor of endothelial apoptosis, blocking this hormone may further predispose to CNS microvascular damage (40).

Taken together, these data are in line with our hypothesis linking miR-29 with SVD and metabolic adverse effects of APDs.

## Discussion

Aggressive behaviors in older patients with dementia require, at times, treatment with APDs to prevent injuries to self or others. However, clinicians often avoid these helpful drugs, as they were associated with CVAE. The hypothesis presented here may allow identification of CVAE vulnerable individuals *via* biomarkers, such as WMHs load and/or high levels of exosomal microRNA-29 in peripheral blood. Aside from presenting putative markers, this hypothesis points to therapeutic targets with potential to restore the function of the brain microvascular bed. Indeed, vascular rehabilitation (VR) is an emerging new field, attempting to resuscitate the integrity of small vessels after strokes, congestive heart failure, or metabolic disorders. As microRNA-29 and the genes it controls are crucial for endothelial health, they represent promising treatment targets for VR (40, 80). Moreover, receptor agonists, mimicking the gene products suppressed by miR-29, may restore brain microvascular function or prevent further loss.

### SIRT-1 Agonists

The addition of SIRT-1 agonist, resveratrol, to elderly individuals on APDs may offer protection against SVD. In cardiac failure, resveratrol was demonstrated to augment the heart function, probably by restoring small vessels’ function (81). Other SIRT-1 agonists were found beneficial in T2DM-associated microvascular damage. For example, SRT1720 was demonstrated to augment microvascular stability and promote wound healing (82, 83). A recent preclinical study revealed that directly lowering miR-29 was beneficial for reversing T2DM and hypercholesterolemia (17).

### VEGF Agonists

Proliferator-activated receptor gamma agonists, known for their ability to induce the expression of VEGF, may provide additional benefit in the prevention of SVD and CVAE (84). Interestingly, a nuclear transcription factor, aryl hydrocarbon receptor (Ahr), a biomolecule directly controlled by miR-29, was documented to upregulate the expression of the VEGF gene in the lung (85). Natural PPAR-gamma agonists, including honokiol, amorfrutin 1, amorfrutin B, amorphastilbol, were demonstrated capable of improving metabolic parameters in animal models of T2DM-associated microvascular damage (86).

### Melatonin Receptor Agonists

Currently used in sleep disorders, these compounds were shown effective in preserving microvascular integrity and have been suggested in the treatment of cerebral ischemia (87). MicroRNA-29b is known to downregulate melatonin-1 receptors, possibly inducing cerebral small vessel lesions (88). As part of the treatment regimen in older individuals on APDs, melatonin may prevent or even restore the integrity of cerebral small vessels.

An interesting concept has been advanced lately regarding the role of adult neurogenesis in various areas of the brain vis-à-vis microvascular integrity and metabolism (89). Interestingly, disruptions of adult neurogenesis were described in depression, anxiety, schizophrenia, and neurodegenerative disorders (90). In addition, it is known that some psychotropic drugs augment the proliferation of neural precursor cells (NPCs) and their differentiation into new neurons (90). As a “metabolic sensor,” SIRT-1 gene was demonstrated to play a key role in adult neurogenesis by directing resources to the energy-consuming NPCs (91). Therefore, augmenting SIRT-1 *via* agonists, such as resveratrol or resveratrol-like agents, may be beneficial for brain neurogenerative niches. For example, the hypothalamic neurogenesis zone is believed to supply new neurons to the medio-basal hypothalamus, an area in charge of metabolism (92–94). Facilitating neuronal replacement in this high wear and tear niche may prove beneficial for insulin/leptin resistance.

A frequently forgotten population are the older individuals with a life-long history of schizophrenia who develop late-life dementia. Because of improved medical care, individuals with schizophrenia live longer and were shown to be at higher risk of late-life dementia compared to the general population (95). Therapeutic guidelines for safe treatment of elderly with chronic schizophrenia and new onset dementia represent an unmet medical need, which will become more prominent along with the demographics of aging population. At present, there are no evidence-based data on the incidence of CVAE risk in this population. Moreover, there is no clear answer to the question: Which individuals require low APDs dosage in order to avoid CVAE and which need and can tolerate higher doses to avoid relapses into psychosis and/or aggressive behavior? In our opinion, biological markers, including WMHs and miR-29, may be valuable diagnostic tools for assessing the CVAE risk in the elderly with schizophrenia and dementia.

## Conclusion

The benefits of elucidating the molecular underpinnings of CVAE and metabolic adverse effects of APDs extend beyond the field of psychiatry and psychopharmacology. Metabolic disorders, cancer, and stroke affect a large percentage of the geriatric population, and their pathogenesis is intertwined with the mechanism of action of APDs. The molecular mechanisms of these conditions superimposed on a background of aging engender a continuum with blurred boundaries between physiology and pathology. Epigenetics of APDs may represent the catalyst for shedding more light into specific areas where further advances are possible, including SVD, cancer, and metabolism.

From the epigenetic perspective, APDs are molecules pertaining to the environment, which trigger specific responses in the body. These responses consist of expression or suppression of genes *via* epigenetic tools, including DNAm and microRNAs. In the case of APDs, these mechanisms may avert cancer at the price of SVD. It is up to future research to provide answers to the following questions: (1) Is it possible to augment the function of the cerebral microvascular bed without triggering cancer? (2) Can agonists of SIRT-1 or VEGF solve this problem without lifting the cell cycle blockade and unleashing malignancies?

## Author Contributions

All authors listed have made substantial, direct, and intellectual contribution to the work and approved it for publication.

## Conflict of Interest Statement

The authors declare that the research was conducted in the absence of any commercial or financial relationships that could be construed as a potential conflict of interest.

## Abbreviations

AD, Alzheimer’s disease; Ahr, aryl hydrocarbon receptor; APDs, antipsychotic drugs; CVAE, cerebrovascular adverse effects; DM, diabetes mellitus; DNAm, DNA methylation; EMPs, extracellular matrix proteins; MicroRNA (miR), microribonucleic acid; NPCs, neural precursor cells; PPAR-gamma, peroxisome proliferator-activated receptor gamma; SIRT-1, NAD-dependent deacetylase sirtuin-1; SVD, small vessel disease; T2DM, type 2 diabetes mellitus; VR, vascular rehabilitation; WMHs, white matter hyperintensities.
